# Investigation on the Flame Retardant Properties and Fracture Toughness of DOPO and Nano-SiO_2_ Modified Epoxy Novolac Resin and Evaluation of Its Combinational Effects

**DOI:** 10.3390/ma12091528

**Published:** 2019-05-10

**Authors:** Markus Häublein, Karin Peter, Gökhan Bakis, Roi Mäkimieni, Volker Altstädt, Martin Möller

**Affiliations:** 1 Department Polymer Engineering, University of Bayreuth, Universitätsstraße 30, 95447 Bayreuth, Germany; markus.haeublein@uni-bayreuth.de; 2 DWI–Leibniz-Institute for Interaktive Materials Aachen, Forckenbeckstraße 50, 52056 Aachen, Germany; peter@dwi.rwth-aachen.de (K.P.); maekimieni@dwi.rwth-aachen.de (R.M.); 3 BASF Polyurethanes GmbH, Elastogranstraße 60, 49448 Lemfoerde, Germany; goekhan.bakis@basf.com; 4 ITMC-Institute of Technical and Macromolecular Chemistry, RWTH Aachen University, Worringer Weg 1, 52074 Aachen, Germany

**Keywords:** epoxy novolac resin, DOPO, nano-SiO_2_, flame retardancy, fracture toughness

## Abstract

In this study, the flame-retardant, thermal and mechanical properties of 9,10-dihydro-9-oxa-10-phosphaphenanthrene-10-oxide (DOPO) and nano-SiO_2_ modified epoxy novolac resin is evaluated, and the combinational effects of both additives are verified. As a hardener, an isophorone diamine (IPDA) and polyetheramine blend is stoichiometrically added to obtain a low viscous epoxy resin system, suitable for resin injection and infusion techniques. The glass transition temperature (Tg) and the silica dispersion quality is affected by the DOPO modification and the nano silica particles. The flame-retardant (FR) and mechanical properties of the additives are investigated separately. The fracture toughness could be increased with the incorporation of both FR additives; however, the effect is deteriorated for higher DOPO amount which is referred to silica particle agglomeration and consequently reduced shear yielding mechanism. Flame-retardant properties, especially the peak heat release rate (pHRR) and the total heat release (THR) could be decreased from 1373.0 kW/m^2^ of neat novolac to 646.6 kW/m^2^ measured by resins with varying phosphorous and silica content. Thermogravimetric analysis (TGA) measurements show the formation of a high temperature stable char layer above 800 °C which is attributed to both additives. Scanning electron microscopy (SEM) images are taken to get deeper information of the flame-retardant mechanism, showing a dense and stable char layer for a certain DOPO silica mixture which restrains the combustible gases from the burning zone in the cone calorimeter test and influences the fire behavior of the epoxy resin.

## 1. Introduction

Since the first patent regarding epoxy and amine systems was submitted in 1934 by Paul Schlack in Germany the importance of resin has gathered increasing relevance. There are several reasons for the wide application range of epoxys nowadays, for instance their good processability with low cure shrinkage and therefore lower residual stresses in the cured part, good mechanical properties and outstanding customization which is verified to the variation of properties with different hardener and resin selection [1]. However, there are also several disadvantages, for example hot-wet and flame-retardant properties, compared to other thermosets, like bismaleimides or phenolic resins which show good intrinsic flame-retardant properties [1].

To overcome the lower flame resistance, different additives like halogenated [2,3] or phosphorous-based [4,5] FRs as well as inorganic fillers (like aluminum trihydroxide ATH) [6] are incorporated into the epoxy matrix and the properties evaluated in the past decades. However, halogenated flame retardants release toxic gases during decomposition and are therefore prohibited by the REACH (Registration, Evaluation, Authorization and Restriction of Chemicals) regulation [7]. Otherwise, inorganic fillers require a high content [6] for the improvement of fire resistance which worsens the processing of the resin system and downgrades its mechanical properties. For these reasons, research in the last decade focuses on phosphorous based epoxy resin systems, especially the 9,10-dihydro-9-oxa-10-phosphaphenanthrene-10-oxide (DOPO) molecule indicates promising flame-retardant properties.

Wang et al. [4] studied the effect of DOPO modified phenolic amine hardeners on the flame-retardant properties of diglycidyl ether of bisphenol A (DGEBA, glass transition temperature, Tg = 171.8 °C) based epoxy resin, showing that 0.83 wt.% phosphorous in the overall system sufficient to reach the UL94-V0 rating and to increase the limiting oxygen index (LOI) from 25.3% for DGEBA up to 36.1% for a modified system. Another study by Wang et al. [5] investigated the influence of DOPO modification in the resin backbone. Tetradiglycidyl diaminodiphenyl methane (TGDDM, Tg = 249 °C) was modified with DOPO and as a hardener 4,4′-diaminodiphenyl sulfone (DDS) was added. The UL94-V0 rating could be reached with only 0.8 wt.% phosphorous and the LOI is increased from 29.1% up to 31.8% for the modified system.

DOPO modified epoxy resin as one component of a flame retarding synergistic system consisting of silica as second component, was investigated by Zhang et al. in 2012 [8]. A DGEBA-m-Phenylendiamine (m-PDA) composite containing oligomeric silsesquioxane (OPS) and DOPO was prepared, while the overall FR-content was kept constant at 5 wt.%. The lowest LOI was found for the composite with 1.25 wt.% OPS and 3.75 wt.% DOPO (LOI = 30.3%), the UL94-V1 rating was verified for every combined system, whereas the lowest peak heat release rate (p-HRR) was determined for 2.5 wt.% OPS and DOPO (p-HRR = 603 kW/m^2^) content. The synergistic effect of the system was verified for a DOPO and silica modified system and justified with the blowing out effect.

However, the synergistic effect of phosphorus-based FR and nano-SiO_2_ particles as a silicon containing additive and the effectiveness in a low Tg-epoxy resins as well as the influence of the flame retardants on the mechanical properties of the epoxy resin system has not been investigated yet. In this study, the fracture toughness, decomposition behavior and flame-retardant properties (Thermogravimetric analysis: TGA and cone calorimeter) are studied for a nano-SiO_2_ and DOPO containing epoxy novolac resin system. In addition, the synergistic effect is discussed, and the structure-property relationship established with scanning and transmission electron microscopy images.

## 2. Materials and Methods

### 2.1. Materials

**Figure 1:**
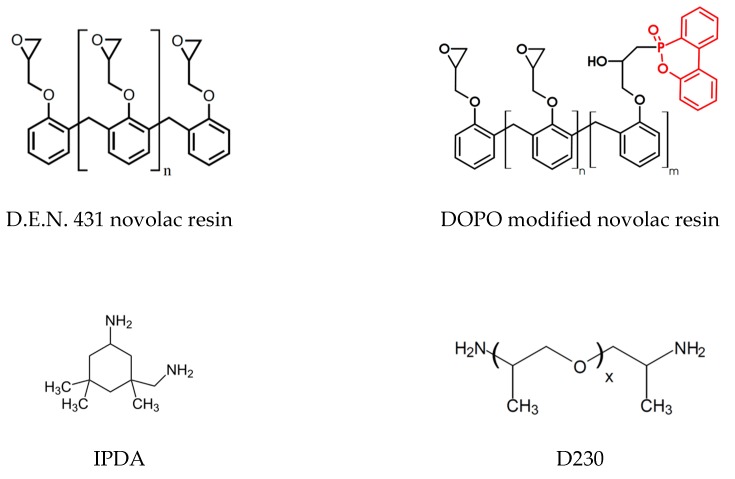
Chemical structure of the epoxy novolac resin system (DOPO modification is highlighted red) and the hardener (blend ratio 50:50 parts by weight).

Epoxy novolac resin D.E.N. 431 (epoxy equivalent weight 176 g/mol) is purchased from Olin. To reduce overall viscosity of the novolac resin 5 wt.% (based on the D.E.N. 431 content) of the reactive diluent Heloxy modifier BD (Hexion, Columbus, OH, USA, epoxy equivalent weight 124 g/mol) is added. As a curing agent isophorone diamine (IPDA, Aradur 42, Huntsman, TX, USA, amino equivalent weight 42 g/mol) and the polyetheramine D230 (Jeffamine XB3403, Huntsman, TX, USA, amino equivalent weight 60 g/mol) in the ratio 50:50 by weight were used. As a flame retardant (FR), 9,10-dihydro-9-oxa-10-phosphaphenanthrene-10-oxide (DOPO) modified epoxy novolac (Struktol VP 3760, Schill&Seilacher, Hamburg, Germany, epoxy equivalent weight 340 g/mol) and spherically shaped nano-SiO_2_-particles as an epoxy novolac master batch (NANOPOX F700, Evonic Industries, Essen, Germany, epoxy equivalent weight 310 g/mol) were added in varying amounts. The chemical structure of the different materials is shown in Figure 1.

### 2.2. Methods

The epoxy novolac resin are weighted and mixed at room temperature with 1700 rpm using a DAC 150.1 FVZ speedmixer. The mixture is degassed in a vacuum chamber. Different amount of FR is generated by replacing some of the D.E.N. 431 by the DOPO- and/or nano-SiO_2_-modified novolac master batches. For the mixture with 1 wt.%, 2 wt.% and 3 wt.% phosphorous (Novolac + 1 wt.% P, Novolac + 2 wt.% P and Novolac + 3 wt.% P), 1/6, 1/3 and 1/2 of the resin is replaced by the DOPO-master batch, respectively. After the stoichiometric amount of the hardener blend is added, the mixture is homogenized with the speedmixer and degassed again, to achieve neat resin plates without air inclusions. The liquid resins system is moulded in a steel plate, which is pre-treated with the release agent Loctite Frekote 770NC to ensure demould, and fully cured at 80 °C for 60 min and 140 °C for 90 minutes (heating rate: 5 K/min) in an oven.

Dynamic mechanical thermo analysis (DMTA) is performed according to DIN EN ISO 6721-2 with a Rheometric Scientific RDA III, the frequency is set to 1 Hz and the deformation to 0.1%. Glass transition temperature is determined by storage modulus (G′) onset, two samples are tested for each material.

The fracture toughness of the material systems is evaluated by the critical stress intensity factor in mode I (K_Ic_-value) according to ISO 13586 with a universal testing machine Zwick Z050 (Zwick Roell, Ulm, Germany). The initial crack is performed by tapping a razor blade into the notch. The initial load is set to 2 N and the testing speed to 10 mm/min, five specimens are tested. The critical energy release rate in mode I loading (G_Ic_-value) is calculated using the Young’s modulus (E) and the following Equation (1) [9]. The modulus was determined with tensile tests of the materials.

G_Ic_ = K_Ic_^2^/E, (1)

TGA measurements using a Netzsch 209 F1 libra (Netzsch, Selb, Germany) are performed under air conditions to evaluate the degradation behavior and the silica content of the material systems. Samples with the weight of 10 to 25 mg are used and heated up from room temperature to 1000 °C with 10 K/min.

Flame retardant properties are investigated with a cone calorimeter iCone (Fire Testing technology FTT, East Grinstead, UK) with the heat flux of 50 kW/m^2^ and a distance between heater and sample of 60 mm. Three samples are tested for each material. The discussed values were averaged, and the standard deviation calculated, one curve is shown representatively in the graphs.

For Raman measurements, a WITec alpha 300 RA+ system equipment with a UHTS 300 spectrometer and a back-illuminated Andor Newton 970 EMCCD camera (Oxford Instruments, Abingdon, UK) was used. The measurements were conducted employing an excitation wavelength of λ = 532 nm and the spectra were recorded with an integration time of 1 s and 50 accumulations (50 x LWD objective, NA = 0.55). All spectra were subjected to a cosmic ray removal routine and baseline correction (software WITec Project FIVE, version 5.1, WITec, Ulm, Germany).

To study the toughening mechanism and the flame-retardant effect, scanning electron microscopy (SEM) using a Zeiss Leo 1530 instrument (Carl Zeiss, Oberkochen, Deutschland) at an acceleration voltage of 3 kV and transmission electron microscopy (TEM) using a Zeiss EM 922 Omega (Carl Zeiss, Oberkochen, Deutschland) with 200 kV acceleration voltage are performed. For SEM studies the sample is coated with a Cressington Sputter Coater to create a 1.3 nm thick conductive platinum layer; images were taken with the in lens and the secondary electron detector. Sample preparation for TEM analysis are performed with an ultra-microtome (Leica EM UC 7, Leica Camera, Wetzlar, Germany) at room temperature. The resulting 50 nm thick samples are placed on a carbon coated copper grid.

## 3. Results and Discussion

### 3.1. Effect of DOPO on Epoxy Resin Properties

Glass transition temperature and fracture toughness of DOPO modified epoxy resin systems can be seen in Table 1. The glass transition temperature shows decreasing values for the DOPO-modified system as the Tg decreases from 109.8 °C for neat novolac to 66.5 °C for 3 wt.% phosphorous containing resin. This change is given by the chemical structure of the DOPO-modified system; the DOPO molecule is chemically bound to the epoxy group of the novolac (see Figure 1) which is consequently not involved in crosslinking and network formation of the cured resin system for that reason [10,11]. The fracture toughness of the systems shows a contrary trend as it seems to be increasing for higher amount of DOPO. Neat novolac reference shows a G_Ic_-value of 109.2 J/m^2^, whereas the highest value (131.8 J/m^2^) is measured with 3 wt.% of phosphorous, but considering standard deviation, they are in the same range.

**Figure 2:**
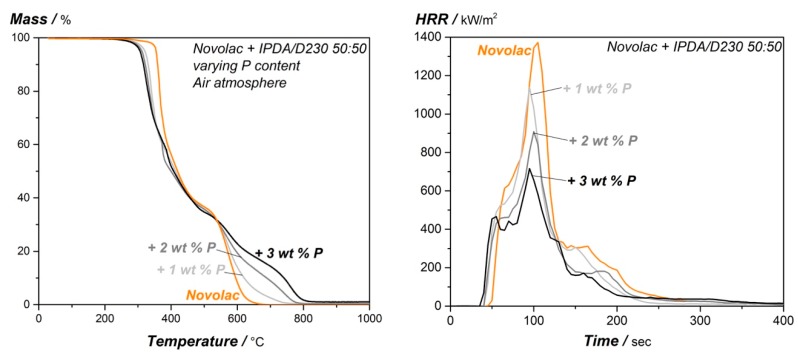
TGA measurement under air atmosphere (**left**) and cone calorimeter test (**right**; 50 kW/m^2^ and distance sample to heater: 60 mm) of the DOPO modified epoxy resin.

The flame-retardant properties of the DOPO modified epoxy resin systems were evaluated with TGA under air conditions and cone calorimeter experiments to study the decomposition and burning behavior. The mass loss rate from the TGA and the heat release rate from the cone calorimeter measurement of the modified systems is shown in Figure 2, a summary of the relevant parameters from TGA and cone is given in Table 2. It is represented in the TGA and cone calorimeter curves that the DOPO additive improve the flame retardancy of the resin system. First, it enhances the char yield at higher temperature, which can be seen in Figure 2 on the left side and in Table 2 since it increases with higher phosphorous content (residual mass @700 °C and @900 °C, see Table 2). The energy release (peak heat release, pHRR and total heat release, THR) is also improved with increasing DOPO content, for highest phosphorous amount, the pHRR and the THR can be reduced from 1373.0 kW/m^2^ and 87.7 MJ/m^2^ for neat novolac to 689.2 kW/m^2^ and 57.4 MJ/m^2^. The reduction in heat release is referred to the gas phase since phosphorous containing flame retardants decompose and release phosphorous-oxide radicals to reduce overall heat release. However, the systems start to decompose earlier for higher phosphorous content, as the T_d5%_-value (the temperature when 5% of the systems is degraded) as well as the time to ignition (tti) are reduced. It is estimated that the resin absorbs energy and starts to decompose earlier, as the glass transition temperature and network density is reduced with a higher amount of phosphorous.

### 3.2. Effect of Nano-SiO_2_ on Epoxy Resin Properties

The influence of silica particles on glass transition and fracture toughness is summarized in Table 3. It is shown that the Tg is increased for the nano-SiO_2_ modified novolac resin which is referred to the surface modification of the silica particles. It is expected that the surface modified particles show high interaction with the epoxy resin which results in an ordered structure of the epoxy resin matrix around the particles. The ordered particle-matrix interphase needs higher thermal energy to start the polymer chain vibration. The compatibility of the commercial NANOPOX product with epoxy resin systems is already discussed in the literature and demonstrated by increasing Tg [12]. In addition, the fracture toughness is also improved for higher silica content from 109.2 J/m^2^ for neat novolac to 207.6 J/m^2^ for 3.9 vol.% SiO_2_ particles. The toughening mechanism for nano silica particles is discussed in the literature already and basically referred to the shear yielding mechanism, a deeper evaluation of toughening effect for the systems given here will be discussed in Section 3.3.

**Figure 3:**
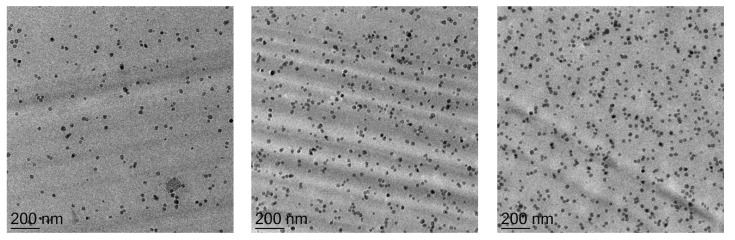
TEM images of nano-SiO_2_ modifed epoxy resin: 0.8 vol.% (**left**), 2.3 vol.% (**middle**) and 3.9 vol.% (**right**).

TEM images are taken to evaluate the dispersion quality of the silica nano-particles and to ensure that cured epoxy resins only show slight amount of small agglomerates. The TEM images are represented in Figure 3 for lowest and highest nano-SiO_2_ content and 2.3 vol.% SiO_2_. It can be summarized that the particles are homogeneously distributed, and no agglomerates are formed even at higher content.

**Figure 4:**
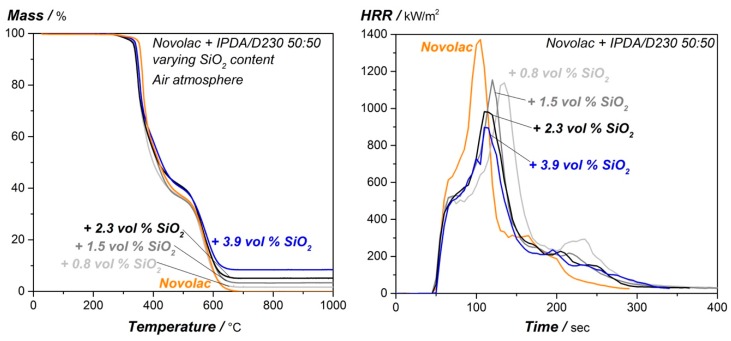
TGA measurement under air atmosphere (**left**) and cone calorimeter test (**right**; 50 kW/m^2^ and distance sample to heater: 60 mm) of the silica modified epoxy resin.

Decomposition and burning behavior of nano-silica modified epoxy resin is shown in Figure 4 and summarized in Table 4. The decomposition behavior on the left side of Figure 4 is only influenced by the silica particles, the residual mass at higher temperature is basically represented by the adjusted silica amount, the T_d5%_-value is reduced for the silica nanocomposites to 338.5 °C and 343.2 °C as highest and lowest value respectively, however the effect is only marginal. Silica amount shows only slight impact on the heat release values and time to ignition from the cone calorimeter experiments. Whereas the peak heat release rate is continuously decreasing from 1373.0 kW/m^2^ for the neat resin to 910.9 kW/m^2^ for a resin containing 3.9 vol.% SiO_2_, the total heat release is decreased for highest silica content. However, the THR increases for lower nano SiO_2_ amount which can be explained by a supportive effect of the particles as they raise the effective surface for the flame. The reduction in THR and especially pHRR can be explained by the amount of non-flammable silica particles. The time to ignition is unaffected for the tested nanocomposites considering standard deviation. It can be summarized that nano-SiO_2_ shows only a slight impact on the flame-retardant properties of the epoxy resin, especially for lower particle loading, compared to the DOPO modified systems for instance.

**Figure 5:**
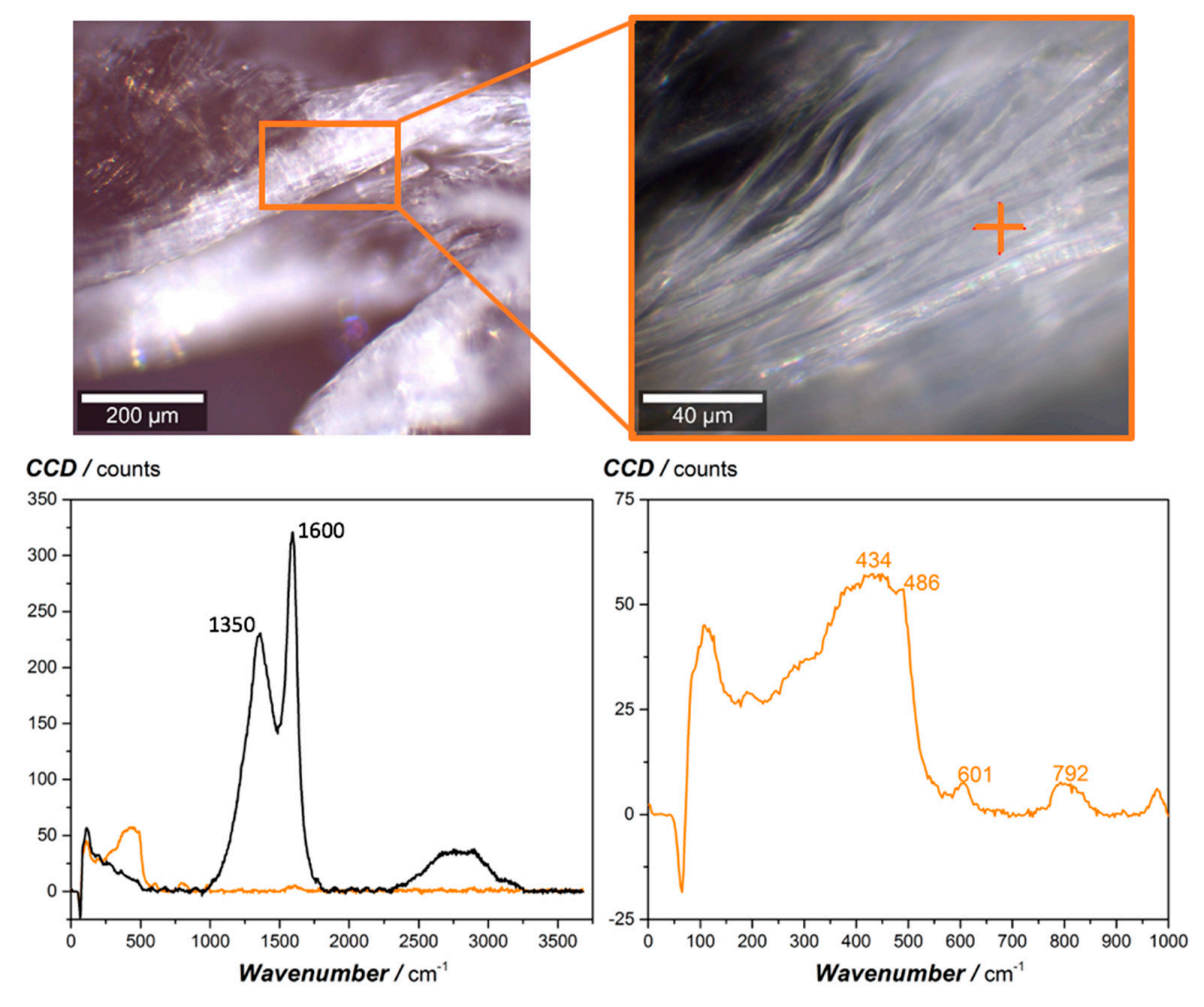
Optical microscopy images of the residual cone calorimeter char of the sample with 2.3 vol.% SiO_2_ with lower and higher magnification and the corresponding Raman spectrum taken at the dense, white appearing layer on top of the burning zone (orange spectrum, measurement position indicated by orange cross in higher magnified microscopy image). The black Raman spectrum was recorded from the inside (opposite side of the burning zone) of the cone colorimeter sample.

The residual cone calorimeter char layer was investigated with Raman spectroscopy to evaluate the flame-retardant effect of the silicon-oxide particles. The optical microscopy images (10× and 50× magnification) taken at the burning zone and the Raman spectra of the sample with 2.3 vol.% SiO_2_ are presented in Figure 5. The orange cross indicates the position, where the Raman spectrum was recorded. The microscopy images reveal that the nano-SiO_2_ particles generate a white appearing, dense and closed glass layer, which results during the burning process of the matrix material. The layer is composed of silicon oxide basically, since the Raman spectrum shows the typical SiO_2_ peaks discussed in the literature [13]. In contrast to this, the black Raman spectrum, which was recorded at the inside (the opposite side to the burning zone) of the cone calorimeter sample, indicates the peaks resulting from carbon black, according to Pawlyta et al. [14]. The different Raman spectra from the inside and the outside (burning zone side) of the nano-SiO_2_ modified sample show that silicon oxide accumulates at the burning side of the sample and generates a dense and closed layer.

### 3.3. Additive Combination: Effect on Glass Transition Temperature, Dispersion Quality and Fracture Toughness Modelling of Modified Systems

**Figure 6:**
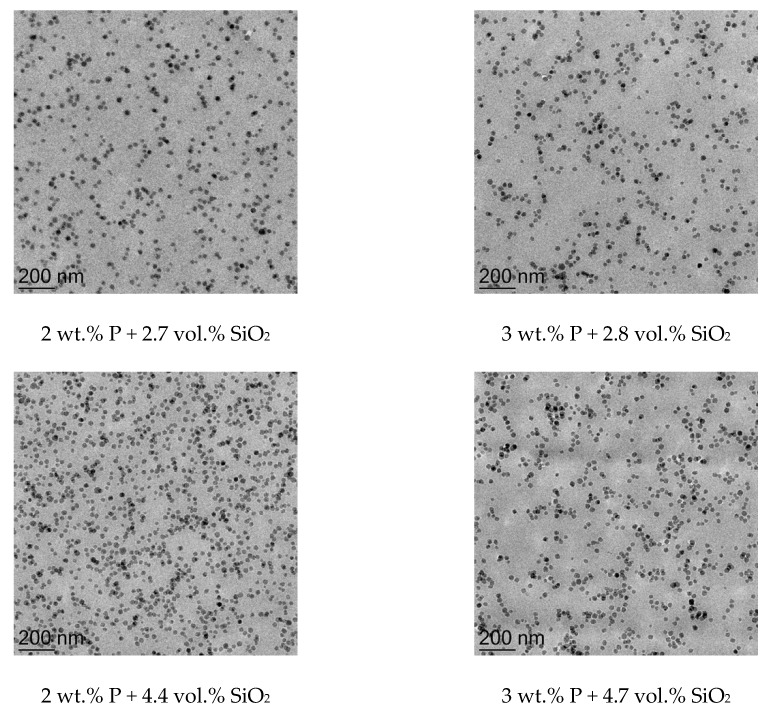
Transmission electron microscopy images of composites with 2 wt.% (**left**) and 3 wt.% (**right**) phosphorous and comparable silica content.

So far, both additives are investigated and evaluated separately; next, the influence on the properties of the combination of both additives will be discussed. The impact on glass transition temperature and fracture toughness is represented in Table 5 and compared to the appropriate system without silica particles. The combination shows a reduction in Tg for 2 wt.% and 3 wt.% phosphorous with higher silica amount. For 2 wt.% and 3 wt.% phosphorous, the glass transition is reduced from 81.9 °C to 75.6 °C by adding 4.4 vol.% SiO_2_ for 2 wt.% P and from 66.5 °C to 62.2 °C for 3 wt.% phosphorous and 4.7 vole% silica. It is represented in the TEM microscopy images in Figure 6 that the dispersion quality of the nano-silica particles is deteriorated for material systems with higher DOPO amount as there are some particle agglomerates visible in the micrographs. Due to agglomeration of silica particles, the particle-matrix interphase is weakened, resulting in continuously decreasing T_g_ for higher SiO_2_-content. The reason for lower dispersion quality is estimated to occur due to the shift of the chemical potential of the liquid resin due to the DOPO modified novolac which is not optimized for the commercial silica particle modification. The particle-particle interaction is estimated to be higher leading to increased agglomerate number.

**Figure 7:**
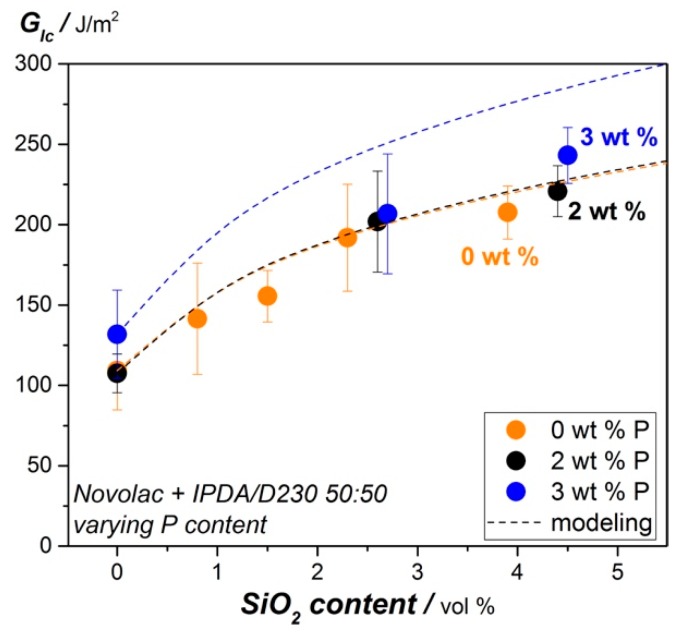
Fracture toughness of the tested material systems.

The G_Ic_-value from Table 5 for the combined systems is plotted in Figure 6 for varying silica content. It can be seen that the nano silica particles show high impact on fracture toughness of modified epoxy resin, increasing from 107.3 J/m^2^ and 131.8 J/m^2^ for 2 wt.% and 3 wt.% P up to 220.8 J/m^2^ (2 wt.% P) and 243.1 J/m^2^ (3 wt.% P) for 4.4 vol.% and 4.7 vol.% SiO_2_ respectively. In addition, the dotted lines in Figure 7 represent a model of the toughening effect of the shear yielding mechanism for nano-sized particles in epoxy resin systems proposed by Hsieh et al. and Johnsen et. al. [15,16]. A particle size of 20 nm is assumed for the calculation according to the datasheet of the supplier of silica particles. It is shown that the modelling fits to the measured values for 0 wt.% phosphorous and the 2 wt.% phosphorous epoxy resin system. This confirms that the dominating toughening mechanisms is shear yielding of 20 nm sized SiO_2_-particles. However, the toughening contribution from shear yielding induced by nano-SiO_2_ particles is overestimated with the modelling for the epoxy resin with 3 wt.% phosphorous. As it is shown in the TEM micrographs in Figure 6, the DOPO modification seems to support particle agglomeration. The overall average particle size is increased for this reason and consequently the shear yielding mechanisms is deteriorated as higher particle sizes lead to lower fracture toughness for this model.

To get deeper information of the fracture and toughening mechanism, the fracture surface is studied with SEM (see Figure 7). For comparison, the fracture surface of neat novolac resin and the fracture surface of only silica modified epoxy resin with comparable nano-SiO_2_ content is represented. For the neat novolac reference a flat and smooth fracture surface is developing during crack propagation which indicates the brittle character of the unmodified system and the low G_Ic_-value of only 109.2 J/m^2^. However, there are also some river lines visible on the fracture surface of the epoxy resin, indicating plastic deformation and shear yielding mechanism.

**Figure 8:**
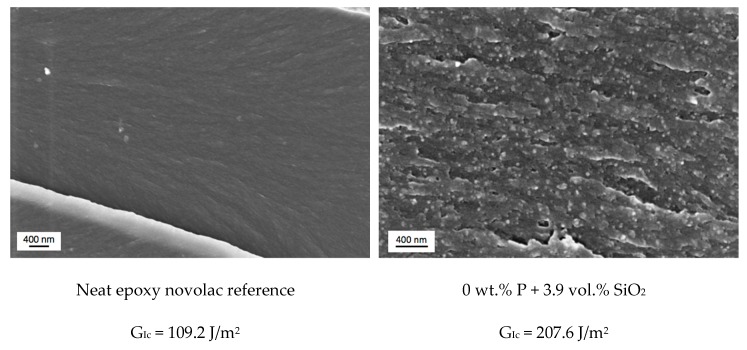

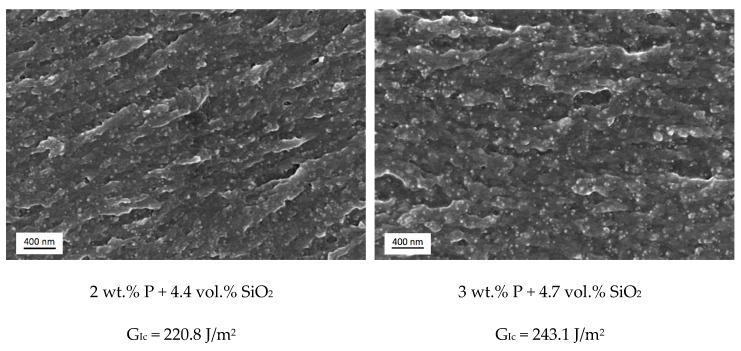
SEM images of K_Ic_-fracture surface of neat novolac (up left), novolac + 3.9 vol.% SiO_2_ (up right) novolac + 2 wt.% phosphorous + 4.4 vol.% SiO_2_ (down left) and novolac + 3 wt.% phosphorous + 4.7 vol.% SiO_2_ (down right). Crack propagation direction: left to right.

For rigid nano silica epoxy resin, different toughening mechanism are already discussed in the literature [17,18,19], these are, in general: shear yielding and particle pull-out and plastic void growth of the matrix [17,18,19]. In Figure 8, for the only silica modified system, the different toughening effects are demonstrated on the fracture surface. However, the particle pull-out toughening mechanism could hardly be verified for the presented systems. Shear yielding seems to be the dominating toughening mechanisms for the system presented here. The river like structure on the fracture surface of silica modified systems in Figure 8 is reflected to the shear yielding mechanism and plastic deformation of the matrix which is more dominant and effective for the SiO_2_ modified epoxy resin compared to neat novolac resin. For the DOPO modified epoxy resins, the river like structure is still pronounced on the surface, especially for the 2 wt.% phosphorous. However, the tendency of silica particles to agglomerate for higher phosphorous content seems to reduce plastic deformation and shear yielding mechanism as the river like structure is less dominant compared to lower phosphorous content. The deteriorating effect has already been discussed previously for the modelling studies (see Figure 7).

### 3.4. Additive Combination: Flame Retardancy of DOPO and Silica Modified Novolac Resin

**Figure 9:**
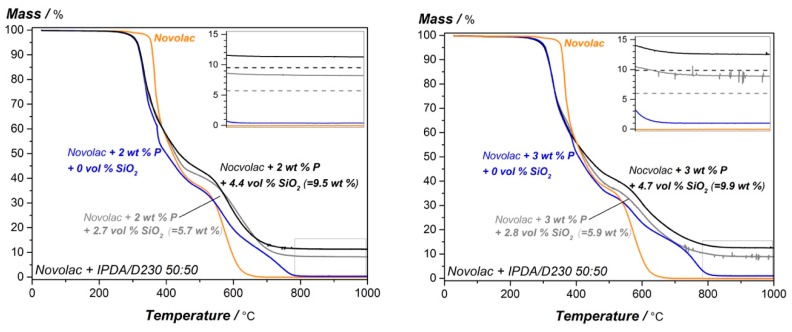
TGA mass loss curve for the modified epoxy novolac resin systems. Influence of silica content on 2 wt.% (**left**) and 3 wt.% (**right**) phosphorous modified resin.

To evaluate the degradation behavior and flame retarding effects of the DOPO and silica modified system, TGA (air atmosphere) and cone calorimeter measurements are performed. The resulting TGA mass loss curves for the reference system without DOPO, with 2 wt.% and 3 wt.% phosphorous and varying silica content are given in Figure 9, the summary (T_d5%_, residual mass @700 °C and @900 °C) is given in Table 6.

The silica content does not show any influence on the decomposition behavior of the novolac epoxy resin at lower temperatures for the DOPO modified systems. The T_d5%_ is nearly unaffected, compared to the appropriate only DOPO modified system. Compared to this, the residual mass at higher temperature (900 °C) is affected by the combination of DOPO and silica. To quantify this in detail, the deviation in between the adjusted silica content and the measured residual mass of the systems at 900 °C (∆m_900°C_) is calculated with the following equation.

∆m_900°C_ = (m_DOPO+SiO2900°C_ − m_DOPO900°C_ − m_SiO2_)/(m_DOPO900°C_ + m_SiO2_), (2)

Equation (2) includes the char yield of the appropriate only DOPO modified epoxy resin at 900 °C (m_DOPO900°C_ from Table 6), the adjusted silica content (m_SiO2_ from Table 6) and the measured residual mass of the nano-silica and phosphorous modified novolac resin (m_DOPO+SiO2900°C_: char yield at 900 °C for DOPO and silica modified system from Table 6).

The deviation is graphically represented in Figure 9 as mismatch between the measured TGA-curve and the adjusted SiO_2_ content (dotted lines) for the combined systems. If the flame retardants would not interact with each other, the deviation should be 0.0% which means that the residual mass is equal to the adjusted silica content. However, as there is a higher deviation observed, it is estimated that a residual char product is generated being composed of both flame-retardant additive types (phosphorous and silica) having high temperature stability as it is stable in the measured area up to 1000 °C. The high temperature stability of the residual product also shows that it is highly oxidized as there is no further degradation and mass loss in air atmosphere. The deviation is in the range of 14–15% for higher and up to 30% and 36% for lower silica content, the biggest difference is given for the 2 wt.% phosphorous and 2.7 vol.% SiO_2_ modified system.

**Figure 10:**
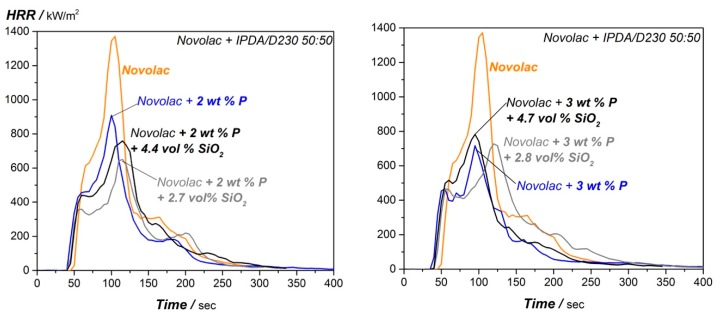
Cone calorimeter measurements for the modified epoxy novolac resin systems. Influence of silica content on 2 wt.% (**left**) and 3 wt.% (**right**) phosphorous modified resin.

To get deeper information about the flammability and decomposition behavior of the material, cone calorimeter tests are performed. The resulting HRR curves are shown in Figure 10 and summarized in Table 7. It is shown that the combination of additives continuously increases the pHRR for the epoxy novolac resin with 3 wt.% phosphorous from 689.2 kW/m^2^ to 814.2 kW/m^2^, further the total heat release is not improved for these systems. However, the time to ignition is nearly unaffected by the combination of DOPO and silica. For the novolac resin with 2 wt.% phosphorous and different silica content, the p-HRR shows lowest value (646.6 kW/m^2^) for 2 wt.% phosphorous and 2.7 vol.% SiO_2_. For higher silica content, the HRR increases again, this tendency is also reflected for the THR. The time to ignition is in addition elevated for the systems with higher silica content and 2 wt.% P.

**Figure 11:**
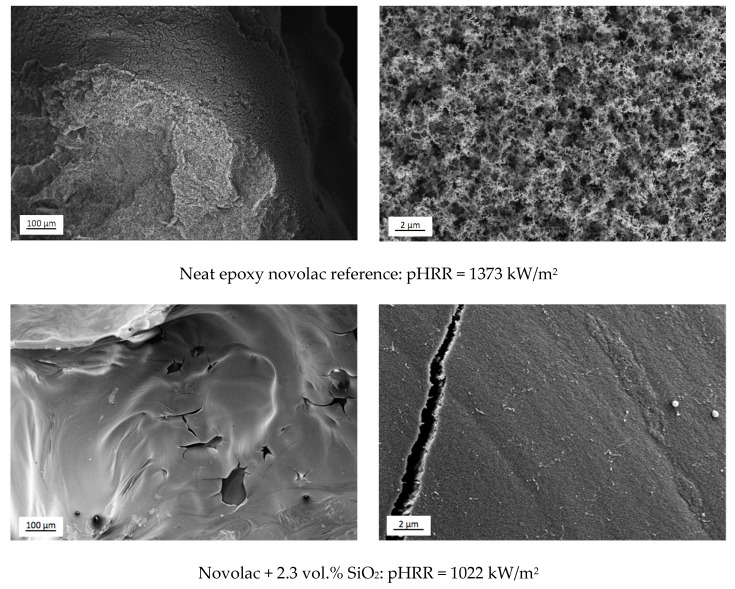

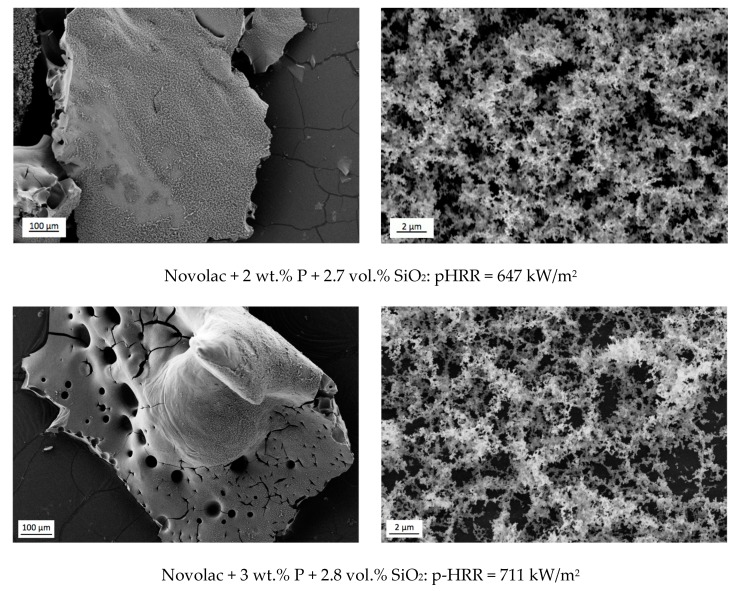
SEM images of the char morphology of cone calorimeter samples from neat novolac, 2.3 vol.% SiO_2_, 2 wt.% phosphorous + 2.7 vol.% SiO_2_ and 3 wt.% phosphorous + 2.8 vol.% SiO_2_) modified resin, with lower (**left**) and higher magnification (**right**).

For deeper evaluation and understanding of the flame retarding effect of the silica and/or DOPO modified epoxy novolac systems, SEM studies of the char morphology of cone calorimeter samples are performed. The images for neat novolac, 2.3 vol.% SiO_2_, 2 wt.% phosphorous + 2.7 vol.% SiO_2_ and 3 wt.% phosphorous + 2.8 vol.% SiO_2_ modified epoxy resin are represented in Figure 11 with different magnifications. The neat novolac char morphology shows rough and uneven surface (see Figure 11, upper image), and a typical carbon black like char residue structure is represented in the image with higher magnification. The morphology shows no flame retarding structure like stable char layer for instance which is also indicated in the HRR-curve in Figure 10 as the curve rises up to the maximum when the majority of the material is already decomposed and afterwards slowly declines again. DOPO modified epoxy resin shows the same char morphology as neat novolac, the flame-retardant effect is verified by the gas phase. However, the 2.3 vol.% silica modified epoxy resin shows smooth char layer with a lower number of leaks. It is estimated that silica particles induce the formation of a char layer which traps the evaporating material up to the point when the inner pressure is reaching the strength of the layer and the combustible gases escape from the generated leaks in the layer. The stable and dense SiO_2_-layer is already proven with the Raman measurement.

The char morphology of cone calorimeter samples with both flame-retardant additives also show the carbon black like structure at higher magnification (see Figure 11), indeed there is a dense layer visible below this morphology which is shown in the image with lower magnification. However, the 3 wt.% phosphorous and 2.8 vol.% SiO_2_ modified novolac resin also shows a high number of leaks in the char layer resulting in decreased flame-retardant properties. In contrast to this, 2 wt.% phosphorous and 2.7 vol.% SiO_2_ modified novolac resin, the char layer seems to be more stable as there are less leaks generated by the combusting gases shown in the SEM micrograph. The stable char layer is also indicated by the pronounced shoulder formation in the HRR-curve (see Figure 10) as it delays the release of combustible gases to the burning zone and therefore reduces the energy release of the fire. Considering the results from the TGA measurements, the resin system with 2 wt.% P + 2.7 vol.% SiO_2_ shows highest deviation from the adjusted silica content (36.1%, see Table 6) which consequently demonstrates that the highest phosphorous amount is incorporated in the char layer and the thickest layer is generated. The combinational effect of the DOPO- and silica modified systems is referred to a stable and dense char layer which develops during the burning process of the material. The layer can clearly be seen in the SEM micrographs in Figure 11. Due to its high temperature stability (stable up to 1000 °C, see Figure 9), it is estimated that the residual product is highly oxidized and composed of silicone- and phosphorous-oxides, basically. Once the protective effect of the char layer reaches its limits as the gas pressure increases up to a critical value, the gas is released by the formation of slight leaks and the burning process is supported. However, it can be seen from the SEM images and the HRR-curve that protective effect of the char layer of the 2.7 vol.% nano-SiO_2_ and 2 wt.% phosphorous modified resin is the most effective one.

## 4. Conclusions

In this study, a novel flame-retardant system containing DOPO and nano-SiO_2_ modified epoxy novolac resin is evaluated and combinational effects are verified using micrograph images. The thermal, mechanical and flame-retardant properties as well as the dispersion quality, fracture and char morphology are investigated. It is shown that DOPO modified novolac resin supports agglomeration of silica particles and decreases glass transition temperature. However, the fracture toughness is enhanced with the combination of phosphorous and silica which is attributed to plastic deformation and shear yielding of the epoxy matrix which is induced by the silica particles. A higher amount of DOPO leads to the formation of agglomerates which deteriorate the shear yielding mechanisms. The decomposition behavior and flame-retardant properties are evaluated with the TGA and the cone calorimeter. The peak-HRR could be reduced from 1373.0 kW/m^2^ for neat novolac up to 646.6 kW/m^2^ for 2 wt.% phosphorous and 2.7 vol.% SiO_2_ modified novolac resin, the time to ignition is decreased from 54 s to 46 s. It is found that there is an optimal composition for DOPO and nano-silica content regarding HRR curve which could be explained by a stable and highly oxidized char layer separating combustible gases from the burning zone. The layer is attributed to both flame retarding additives which is verified with the TGA curves as the residual mass exceeds the overall mass of the additives at higher temperatures (800–1000 °C) and results in a silicone and phosphorous-oxide containing residual product. The char structure could be verified further with SEM micrographs.

## Figures and Tables

**Table 1:** Adjusted phosphorous content, glass transition temperature and fracture toughness of DOPO modified epoxy resin.

	Phosphorous Content/wt.%	Tg/°C	G_Ic_/J/m^2^
Novolac (Reference)	0.0	109.8 ± 0.3	109.2 ± 24.4
Novolac + 1 wt % P	1.0	96.7 ± 0.5	123.8 ± 18.6
Novolac + 2 wt % P	2.0	81.9 ± 0.9	107.3 ± 12.0
Novolac + 3 wt % P	3.0	66.5 ± 0.6	131.8 ± 27.5

**Table 2:** T_d5%_, residual mass @700 °C and @900 °C from TGA under air atmosphere and time to ignition (tti), peak heat release rate (pHRR) and total heat release (THR) from cone calorimeter experiments for DOPO modified epoxy resins.

	TGA			Cone Calorimeter		
	T_d5%_/°C	Residual Mass		tti/sec	pHRR/kW/m^2^	THR/MJ/m^2^
		@700 °C/wt.%	@900 °C/wt.%			
Novolac (Reference)	354.6	0.1	0.1	53.7 ± 2.9	1373.0 ± 151.6	87.7 ± 1.3
Novolac + 1 wt.% P	320.5	2.5	0.4	45.7 ± 1.5	1131.3 ± 59.8	73.3 ± 4.2
Novolac + 2 wt.% P	311.7	8.9	0.4	42.7 ± 0.6	917.7 ± 43.4	64.6 ± 1.5
Novolac + 3 wt.% P	306.2	14.4	1.0	41.0 ± 2.0	689.2 ± 43.2	57.4 ± 1.1

**Table 3:** Adjusted silica content, glass transition temperature and fracture toughness of nano-SiO_2_ modified epoxy resin.

	Nano-SiO_2_ Content/vol.%	Tg/°C	G_Ic_/J/m^2^
Novolac	0.0	109.8 ± 0.3	109.2 ± 24.4
Novolac + 0.8 SiO_2_	0.8	111.3 ± 1.3	141.5 ± 34.6
Novolac + 1.5 SiO_2_	1.5	111.0 ± 0.5	155.5 ± 16.1
Novolac + 2.3 SiO_2_	2.3	110.1 ± 0.1	191.9 ± 33.2
Novolac + 3.9 SiO_2_	3.9	112.5 ± 0.4	207.6 ± 16.5

**Table 4:** T_d5%_, residual mass @700 °C and @900 °C from TGA under air atmosphere and time to ignition (tti), peak heat release rate (pHRR) and total heat release (THR) from cone calorimeter experiments for nano-SiO_2_ modified epoxy resins.

	TGA			Cone Calorimeter		
	T_d5%_/°C	Residual Mass		tti/sec	pHRR/kW/m^2^	THR/MJ/m^2^
		@700 °C/wt.%	@900 °C/wt.%			
Novolac	354.6	0.1	0.1	53.7 ± 2.9	1373.0 ± 151.6	87.7 ± 1.3
Novolac + 0.8 SiO_2_	339.2	1.7	1.5	53.3 ± 1.2	1138.9 ± 53.3	103.9 ± 3.3
Novolac + 1.5 SiO_2_	338.6	3.3	3.3	52.7 ± 1.5	1141.9 ± 84.4	97.0 ± 2.0
Novolac + 2.3 SiO_2_	338.5	5.1	5.0	52.7 ± 1.2	1022.4 ± 34.7	90.2 ± 1.0
Novolac + 3.9 SiO_2_	343.2	8.6	8.6	54.7 ± 0.6	910.9 ± 58.3	84.4 ± 2.9

**Table 5:** Adjusted silica content, glass transition temperature and fracture toughness of DOPO and nano-SiO_2_ modified epoxy resin.

	Phosphorous Content/wt.%	Nano-SiO_2_ Content/vol.%	Tg/°C	G_Ic_/J/m^2^
Novolac + 2 P	2.0	0.0	81.9 ± 0.9	107.3 ± 12.0
Novolac + 2 P + 2.7 SiO_2_	2.0	2.7	78.2 ± 0.1	201.9 ± 31.4
Novolac + 2 P + 4.4 SiO_2_	2.0	4.4	75.6 ± 0.1	220.8 ± 15.9
Novolac + 3 P	3.0	0.0	66.5 ± 0.6	131.8 ± 27.5
Novolac + 3 P + 2.8 SiO_2_	3.0	2.8	63.8 ± 0.1	206.8 ± 37.3
Novolac + 3 P + 4.7 SiO_2_	3.0	4.7	62.2 ± 0.1	243.1 ± 17.4

**Table 6:** T_d5%_, residual mass @700 °C and @900 °C from TGA curves and deviation according to Equation (2).

	Nano-SiO_2_ Content: m_SiO2_/wt.%	T_d5%_/°C	Residual Mass		Deviation @900 °C: ∆m_900°C_/%
			@700 °C: m_700°C_/wt.%	@900 °C: m_900°C_/wt.%	
Novolac (Reference)	0.0	353.9	0.1	0.0	0.0
Novolac + 2 P	0.0	311.7	8.9	0.4	0.0
Novolac + 2 P + 2.7 SiO_2_	5.7	314.0	11.5	8.3	36.1
Novolac + 2 P + 4.4 SiO_2_	9.5	313.0	13.3	11.3	14.1
Novolac + 3 P	0.0	306.2	14.4	1.0	0.0
Novolac + 3 P + 2.8 SiO_2_	5.9	303.1	14.7	9.0	30.4
Novolac + 3 P + 4.7 SiO_2_	9.9	303.1	18.9	12.6	15.6

**Table 7:** Time to ignition (tti) and peak heat release rate (pHRR) from cone calorimeter experiments.

	Nano-SiO_2_ Content/vol.%	tti/sec	pHRR/kW/m^2^	THR/MJ/m^2^
Novolac	0.0	53.7 ± 2.9	1373.0 ± 151.6	87.7 ± 1.3
Novolac + 2 P	0.0	42.7 ± 0.6	917.7 ± 43.4	64.6 ± 1.5
Novolac + 2 P + 2.7 SiO_2_	2.7	46.0 ± 1.0	646.6 ± 54.9	58.3 ± 1.9
Novolac + 2 P + 4.4 SiO_2_	4.4	44.7 ± 0.6	781.0 ± 19.2	73.5 ± 2.7
Novolac + 3 P	0.0	41.0 ± 2.0	689.2 ± 43.2	57.4 ± 1.1
Novolac + 3 P + 2.8 SiO_2_	2.8	44.7 ± 0.6	711.3 ± 30.7	76.9 ± 2.3
Novolac + 3 P + 4.7 SiO_2_	4.7	43.3 ± 0.6	814.2 ± 28.0	63.6 ± 0.6
